# Price Policy and Taxation as Effective Strategies for Tobacco Control

**DOI:** 10.3389/fpubh.2022.851740

**Published:** 2022-04-05

**Authors:** Richard Felsinger, Ernest Groman

**Affiliations:** Department of Social and Preventive Medicine, Center for Public Health, Medical University of Vienna, Vienna, Austria

**Keywords:** tobacco, taxes, price elasticity, consumption, public policy, economics

## Abstract

**Objective:**

Only 13% of the world's population are living in countries imposing appropriate tobacco tax-rates. This study aims to promote the implementation of price policy measures as a striking tobacco control strategy in Austria and to encourage other countries to further increase their taxes to WHO best-practice levels.

**Method:**

This study used the yearly economic data from Austria from 1997 to 2015. Applying a model for regression analysis, the price elasticity of total tobacco consumption was estimated.

**Results:**

Between 1997 and 2015 the price elasticity of demand for tobacco products (including cigarettes, cigars, and other tobaccos) was −0.661, however, the result is statistically insignificant. When excluding 2 anomalous years and removing a variable of the regression model the elasticity was −0.691 and statistically significant, indicating that a 1% increase in tobacco prices will result in a 0.691% decrease of tobacco consumption.

**Conclusion:**

The responsiveness of Austrian smokers to price changes has increased during the last decades. Because other activities showed no significance in the analysis, this study should encourage countries world-wide to use price policy and taxation more intensively in order to reduce smoking rates effectively.

## Introduction

According to the World Health Organization, tobacco is the most preventable cause of death in the world (1). To curb the tobacco epidemic multiple strategies are used such as warning labels on cigarette packages, smoking cessation and smoking bans. Tobacco tax increases are supposed to be one of the most important and most effective tools for reducing cigarette demand, for lowering the percentage of smokers, against smoking initiation, especially among the adolescents, and for decreasing adverse health effects (2–4). Despite the increasing evidence, many countries have extremely low taxes: According to the *WHO report on the global tobacco epidemic 2021*, only 40 countries levy taxes that represent more than 75% of the retail price of a pack of cigarettes in 2020, which is an increase of only two countries since 2018. In other words, only 13% of the world's populatio n are living in countries imposing appropriate tax-rates. That shows that even 13 years after the introduction of MPOWER by the WHO, raising taxes is the measure with the least adoption and improvement (5). The acronym MPOWER describes a package of six measures assisting Parties of the WHO Framework Convention on Tobacco Control (WHO-FCTC) in the implementation of specific WHO-FCTC provisions to reduce the demand for tobacco. The six components are: Monitoring tobacco use and prevention policies, Protecting people from tobacco smoke, Offering help to quit tobacco use, Warning about the dangers of tobacco, Enforcing bans on tobacco advertising, promotion and sponsorship and Raising tobacco taxes (1).

A review published in 2012 confirms that price policy is a striking tobacco control strategy with the greatest impact on young and poor people and that contrary statements about adverse economic effects are overstated or false (6). In fact, there is much evidence that tobacco tax increases can generate additional fiscal revenues, reducing tobacco-related illness and death simultaneously (2).

Austria's total tax share (inclusive VAT; in percent of the weighted average price) hardly changed during the last years and is about 77% (7). Being a Member State of the EU, Austria applies a mixed structure, constantly reducing the share of ad valorem tax and increasing that of specific tax. As of April 1st 2021, the specific excise is €68.00 per 1,000 cigarettes, the ad valorem excise 34.5% and as of April 1st 2022 the specific excise tax will be increased to €73.00, the ad valorem excise tax will be decreased to 33% (8).

Price elasticity, regarding the consumption of cigarettes and tobacco products, respectively, describes the percentage change in tobaccos demanded in response to a 1% change in price. Many studies have been conducted in different high-income countries to investigate this price elasticity and show similar results in the range of 0.25 to 0.5 (0.4 on average) (2, 6), thus tobacco consumption is *price inelastic*: e.g., Canada (−0.45 to −0.47) (9), Italy (−0.43) (10), Argentina (−0.31) (11).

In the last years there have been investigations in low-income and middle-income countries too, but the situation does not seem to be clear yet. Some studies claim that demand of cigarettes is likely to be as or even more responsive to price changes than it is in high-income countries, but others show the opposite (6, 12). Overall, the estimated price-elasticities vary within a wide range with an averaged value of about 0.5 in low- and middle income countries (5).

Wörgötter and Kunze studied the effect of price policy on cigarette demand and described tobacco product prices relative to those of other consumer goods in Austria between 1955 and 1983. The results demonstrated that Austrian tobacco consumption is price inelastic and independent of general consumption patterns. A 1% increase of cigarette prices leads to a decrease of cigarette consumption by 0.54%. Therefore, tobacco tax increases would not cause a reduction of tax revenues (13). More recent studies concerning this topic, as they are available in many other countries, do not exist in Austria.

17.3% of Austrian men and about 17% of Austrian women smoke daily or almost daily (14). Considering the high prevalence of smoking tobacco use of 32% among 15–24 year old Austrians, which is considerably higher compared to several other European countries (21.4% in Central Europe, Eastern Europe, and Central Asia) (15), it is absolutely necessary to call attention to this problem as well as to the fact that tax increases are without much doubt effective strategies to reduce smoking prevalence among minors.

## Methods

### Data

The study population is the population of Austria. The necessary data were obtained from *Statistik Austria* (*STATcube*, the statistical database system of *Statistik Austria*, was utilized). Following Wörgötter and Kunze this study uses the following yearly economic data from Austria from 1997 to 2015:

- consumption expenditure of all tobacco products at constant prices of 1996
- Consumer Price Index
- Wholesale Price Index of tobacco products
- real consumption expenditure by private households in million Euros

Additionally, data about the annual average population of Austria is used.

The Consumer Price Index (CPI) is an economic indicator which measures price-changes and inflation over time. It is based on a representative selection of goods and services purchased by an average Austrian household (basket of consumer goods) and is published by *Statistik Austria* every month. In order to compare index values of different base years and to continue previous indices appropriate chaining factors are used. The therewith continued series of the “CPI 1996” (base year 1996) is published by Statistic Austria.

The Wholesale Price Index (WPI) displays the price trend of goods disposed by the wholesale trade. This study uses the wholesale price index of tobacco products (PITP). The basket of goods of this price index contains the following items: cigarettes (96%), cigars (1%), and tobaccos (3%). Because of several revisions a reasonable comparison of index values of different base years is possible only when using chaining factors in order to continue the series of the “WPI of tobacco products 1996” (base year 1996) till 2015.

According to the Austrian Tobacco Tax Act 1995 (§ 9), tax liability arises when transporting tobacco products in (fiscal) free circulation. By definition, this is the case when taking tobacco products from the tax warehouse or when producing tobacco products. Thus, tobacco taxes have to be paid by the owner of the tax warehouse or the manufacturer (§ 10) and are levied on the maximum retail selling price (8). Therefore, the wholesale price already includes tobacco duties and consequently it can be assumed that the wholesale price and the retail selling price will increase when raising taxes on tobacco. Accordingly, the wholesale price index of tobacco products can be used to examine the effects of taxation on tobacco consumption.

### Statistical Methods

The major question of the study refers to the price elasticity of tobacco consumption in Austria between 1997 and 2015. The study uses the following model for regression analysis to answer this question:

$$\begin{array}{l} \text{Δt}{\text{c}}_{\text{t}}=\beta_{0}+\beta_{1}\text{Δr}{\text{p}}_{\text{t}}+\beta_{2}\text{Δtp}{\text{c}}_{\text{t}}+\beta_{3}{\text{D}}_{04}+\beta_{3}{\text{D}}_{09}+\beta_{5}\text{t} \end{array}$$

The dependent variable tc describes the logarithm of the per capita tobacco consumption at constant prices of 1996. The independent/explanatory variables rp and tpc describe the logarithms of relative prices and per capita total private consumption, respectively. D_04_ and D_09_ are dummy variables and refer to political measures taken by the Austrian government to reduce tobacco consumption in 2003 and 2009, respectively. Δ refers to the changes compared with the previous year. The variable t displays a linear trend or rather the time factor (1 in 1997 and 19 in 2015).

Two legislative tobacco control measures were introduced in 2003 and 2009 and are represented in the equation by two dummy variables:

- D_04_: In 2003, Austria implemented the EU Directive 2001/37/EG (regulating the upper limits for certain substances, such as tar, nicotine and carbon monoxide, contained in cigarettes and the labeling of packages of tobacco products, including warning labels and information about ingredients) by assimilating the Tobacco Act. Since September 30, 2003, all packages of tobacco products have to fulfill the EU requirements and have to bear warning texts. Because of the possible effects of warning labels on smoking behavior (see below) (2), it can be assumed that this tobacco control measure reduced cigarette consumption among Austrian smokers in the year after the implementation. However, this effect possibly slackened during the following years because people got used to the warning messages and were better informed about the health consequences of smoking (16). Hence, the variable D_04_ respects the possible effects of this new legislative in 2004 and has a value of 1 in 2004 and 0 in all other years.
- D_09_: In 2008, the government adopted a smoke-free legislation relating to gastronomy. Since January 1, 2009, smoking is prohibited in rooms, where food and beverages are served, including premises, where people are accommodated such as hotels, but with several exceptions such as the permission to smoke in separated rooms. To consider these possible effects the variable D_09_ was added to the model. It can be supposed that the effectiveness of this legislation persisted over the following years and, according to the statistical method of other studies, for example Martinez et al. (11), the dummy variable D_09_ has the value of 1 between 2009 and 2015 and 0 in all other years.

In the following the remaining quoted variables are described more in detail.

- Δtc: To calculate the real value of tobacco consumption at constant prices of 1996 the nominal value of the consumption expenditure of tobacco products in million Euros and the wholesale price index of tobacco products, which are available on *STATcube*, were used.

$$\begin{array}{l} \text{t}{\text{c}}_{\text{t}}\left(\text{real}\right)\;=\;\frac{\text{t}{\text{c}}_{\text{t}}\;\left(\text{nominal}\right)}{\text{PIT}{\text{P}}_{\text{t}}}\;\cdot \;100 \end{array}$$

The consumption expenditure per person was calculated by dividing the real value of tobacco consumption by the annual average Austrian population. Then an index value was generated for the yearly per capita tobacco consumption using 1996 as base year.


$$\begin{array}{l} \text{Index }=\;\frac{\text{t}{\text{c}}_{\text{t}}}{\text{t}{\text{c}}_{1996}}\;\cdot \;100 \end{array}$$

Further, after taking the logarithm of these index values, the absolute changes compared with the previous year were calculated. The resulting data (Δtc) were used in the linear regression model.

- Δtpc: The real value of the total private consumption was calculated by using the nominal value of the consumption expenditure by private households in million Euros and the Consumer Price Index. The subsequent steps equal those described under “Δtc.”
- Δrp: The yearly wholesale price index of tobacco products (PITP) was divided by the yearly consumer price index (CPI) and the result logarithmized. For both price indices the December values were used. Finally, the absolute differences compared with the previous year were calculated.

$$\begin{array}{l} \text{Δrp }=\text{ log }\frac{\text{PIT}{\text{P}}_{\text{t}}}{\text{CP}{\text{I}}_{\text{t}}}-\text{log }\frac{\text{PIT}{\text{P}}_{\text{t}-1}}{\text{CP}{\text{I}}_{\text{t}-\;1}} \end{array}$$

## Results

**Figure 1:**
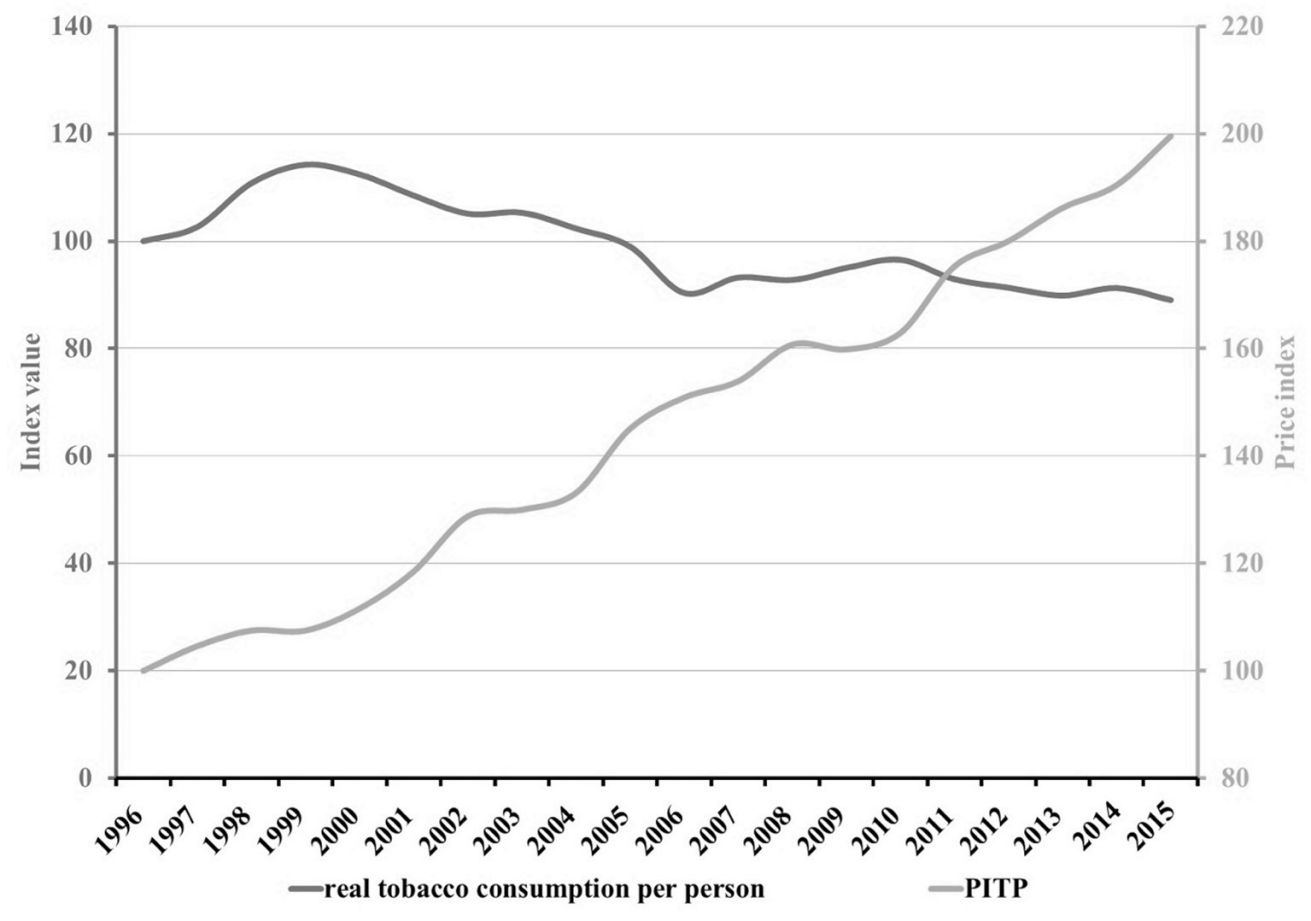
Trends of the consumption expenditure of tobaccos per person and the wholesale price index of tobacco products (PITP) 1996–2015.

Figure 1 shows the trend of the per capita consumption expenditure of tobacco products (index values) as well as that of the wholesale price index of tobacco products (PITP) between 1996 and 2015 in Austria.

Using *IBM SPSS Statistics* all unknown parameters of the defined model are estimated in one multiple regression analysis in order to determine the influence of the independent variables, each adjusted for the other variables. The parameters of the regression analysis are interpreted in the following way: β_1_ describes the influence of relative price changes of tobacco products (Δrp) on relative changes in tobacco consumption (Δtc). β_2_ describes the influence of relative changes in total private consumption (Δtpc) on Δtc. β_3_ and β_4_ stand for the influence of the two dummy variables (D_04_ and D_09_), which represent political measures implemented in the observed time period, on relative changes in tobacco consumption. β_5_ shows to which extend the autonomous growth rate of tobacco consumption (i.e., independent of prices, general consumption patterns, etc.) changes annually. The parameters β_1_ and β_2_ can be interpreted as elasticities.

In the analyses using above data the coefficient of determination was estimated: *R*^2^ = 0.466 and adjusted *R*^2^ = 0.260. This number represents the proportion of the variance in the dependent variable that is predictable from the statistical model. Accordingly, about 50% of the variance in Δtc can be explained by the model. The Durbin-Watson statistic was used to ascertain the presence of autocorrelation in the residuals: DW = 2.168, indicating that there is no autocorrelation.

The results of the multiple regression analysis are presented by Table 1. Coefficient β_1_, typifying the price elasticity, is −0.661. However, this value is not significant at a 5% significance level (Sig. > 0.05). Moreover, the *F*-test is statistically insignificant. Thus, the regression model is statistically not significant on the whole [*F*_(5, 13)_ = 2.266 *p* > 0.05] and its coefficients were not be interpreted.

**Table 1:** Results of the regression analysis for the period 1997–2015.

**Coefficients**					
**Coefficients**		**SD**	**Sig**.	**95% CI**	
				**Lower limit**	**Upper limit**
β_0_	0.021	0.009	0.038	0.001	0.041
β_1_	−0.661	0.313	0.054	−1.337	0.015
β_2_	−0.719	0.812	0.392	−2.474	1.035
β_3_	−0.018	0.015	0.266	−0.050	0.015
β_4_	0.011	0.013	0.408	−0.017	0.039
β_5_	−0.002	0.001	0.090	−0.004	0.000

When looking at Figure 1, it is noticeable that the real tobacco consumption per person increased considerably between 1997 and 1998 although prices were constantly rising, as in all other years. The development in these 2 years seems to be in contrast to the general trend of decreasing tobacco consumption in most other years. It can be concluded that this uptrend in the very beginning of the observed time period is not explainable and it can be assumed that it has nothing to do with the Austrian price policy of tobacco products. The values of these 2 anomalous years may have an adverse impact on the results, which should reflect the general trend seen in the overall period, and may be the cause for its insignificance. Therefore, a separate statistical analysis was conducted for the period 1999 till 2015, without the 2 years with antithetic trends.

Moreover, SPSS provides several methods of entering variables into the model. The first evaluation for the complete time period (see above) used “*Enter*:” In this case SPSS enters all variables at one time. In the following regression analysis for the shorter time period the method “*Backward*” was utilized: Again, SPSS enters all independent variables at one time (Model 1 below) and then removes successively single variables (Model 2–5 below) based on a particular significance level (*F*-value ≥ 0.10). In the conducted analysis SPSS removed the variables in the following order:

D09 (Model 2)

t (Model 3)

D04 (Model 4)

TPC (Model 5)

When using all independent variables (Model 1) the result clearly differs from the previous evaluation. A higher coefficient of determination was estimated as follows: *R*^2^ = 0.579 and adjusted *R*^2^ = 0.388. A larger proportion of the of the variance in the dependent variable is predictable from the amended statistical analysis. However, only coefficient β_1_ is statistically significant and its value is somewhat greater or rather the price elasticity is higher than that in the previous analysis (about −0.69 vs. −0.66). All in all the model remains statistically insignificant. Following the “*Backward*” procedure, SPSS removed the dummy variable D_09_ first. With only the four remaining independent variables (rp, tpc, D_04_, t) the regression model was significant on the whole: [*F*_(4, 12)_ = 4.129 *p* < 0.05]. When examining the coefficients in detail, it becomes obvious that each one, apart from the important coefficient β_1_, representing the price elasticity, remains statistically insignificant. According to this estimation (using a shortened time period and after removing the dummy variable D_09_, i.e., Model 2), coefficient β_1_ and accordingly the price elasticity of the Austrian tobacco consumption is −0.691. Thus, it can be expected that a 1% increase in tobacco prices will result in a 0.691% decrease of tobacco consumption. Regarding coefficient β_2_, a one percent increase of total private consumption leads to a decline of tobacco consumption by 1.158%. However, the coefficient is not statistically significant. The coefficients β_3_ and β_4_ indicate that the political tobacco control strategies, adopted during the observed time period, only marginally influenced smoking behavior. However, both coefficients are not significantly different from zero and the regression model even becomes statistically significant on the whole when removing D_09_. The autonomous component of tobacco consumption growth is supposed to decline by 0.1% per year, but, again, coefficient β_5_ is statistically not significant. The equation for the regression line (Model 2) is estimated as follows:

$$\begin{array}{l} \text{Δt}{\text{c}}_{\text{t}}=0.010-0.691\text{Δr}{\text{p}}_{\text{t}}-1158\text{Δtp}{\text{c}}_{\text{t}}-0.017{\text{D}}_{04}-0.001\text{t} \end{array}$$

The results of Model 1–5 (in condensed form) of this evaluation are shown in Table 2.

**Table 2:** Results of the regression analysis for the period 1999–2015.

**Coefficients**						
**Model**	**Coefficients**		**SD**	**Sig**.	**CI 95%**	
					**Lower limit**	**Upper limit**
1	β_0_	0.010	0.009	0.297	−0.010	0.031
	β_1_	−0.691	0.247	0.018	−1.235	−0.146
	β_2_	−1.158	0.700	0.126	−2.699	0.382
	β_3_	−0.017	0.012	0.180	−0.043	0.009
	β_4_	−5.232E−6	0.011	1.000	−0.024	0.024
	β_5_	−0.001	0.001	0.586	−0.003	0.002
2	β_0_	0.010	0.008	0.237	−0.008	0.028
	β_1_	−0.691	0.230	0.011	−1.191	−0.190
	β_2_	−1.158	0.656	0.103	−2.587	0.271
	β_3_	−0.017	0.011	0.150	−0.041	0.007
	β_5_	−0.001	0.001	0.350	−0.002	0.001
3	β_0_	0.003	0.003	0.392	−0.004	0.010
	β_1_	−0.711	0.228	0.008	−1.204	−0.218
	β_2_	−0.813	0.551	0.164	−2.003	0.376
	β_3_	−0.015	0.011	0.187	−0.038	0.008
4	β_0_	0.002	0.003	0.632	−0.005	0.009
	β_1_	−0.643	0.230	0.014	−1.137	−0.149
	β_2_	−0.837	0.569	0.163	−2.057	0.383
5	β_0_	0.000	0.003	0.966	−0.007	0.007
	β_1_	−0.728	0.231	0.007	−1.221	−0.235

*All independent variables were entered at one time (Model 1). Afterwards single variables were successively removed (Model 2–5) based on a particular significance level (F-value ≥ 0.10)*.

## Discussion

This study examined the price elasticity of tobacco products and the dependency of general consumption patterns in Austria, using yearly economic data during the period 1997 to 2015 and applying a multiple regression analysis. The results are almost comparable with those of Wörgötter and Kunze (13) and with the situation in many other countries (see Section Introduction). Austrian tobacco consumption is still price inelastic. In comparison to similar studies from other developed countries Austria's price elasticity is above average. According to this investigation, nowadays consumers of tobacco products are probably much more price responsive than they have been 30 years ago. A price increase of 1% caused a decrease of tobacco consumption by ~0.54% in the time period 1961–1983 compared to ~0.69% between 1999 and 2015 in Austria. However, the corresponding confidence interval is wide and therefore the true effect is not ascertained: The confidence interval in the regression analysis varies from −1.191 to −0.190. Although this range is quite wide, even at its upper limit the price elasticity is still below zero. Hence, no “perfectly inelastic” or “abnormal elastic” behavior is possible in the model. Possible reasons for the low estimates of the adjusted *R*^2^–indicating the proportion of the variance in the price elasticity which is predictable from the statistical model—are the high number of independent variables together with the relatively low number of cases (i.e., number of years) included in the model. The unexpected increase of tobacco consumption during the years 1997 and 1998 might be a consequence of intensive public information campaigns to combat smuggling by warning the Austrian population about possible high concentrations of unwanted ingredients of illegal manufactured cigarettes (e.g., high concentrations of cadmium and lead, rat feces and even possible radioactive contamination).

Recently, Shuval et al. investigated the association between cigarette prices and smoking behavior in Israel, using retail prices of cigarettes and data from a repeated cross-sectional survey. By combining the elasticities of smoking prevalence and smoking intensity they estimated a total price elasticity of cigarette demand in a range of −0.46 to −0.92, that bears comparison with the results of the present study and underlines the importance of raising tobacco prices to reduce tobacco consumption (17).

Prices of tobacco products are influenced by many different factors, but studies show that higher taxes result in price increases at least in the same proportion because of the oligopolistic structure of the cigarette market and the addictive nature of tobacco products (18, 19). Hence, the results indicate that taxation of tobacco products is a very effective measure to reduce tobacco use in Austria and most probably more effective than other methods such as areal smoking bans or warning labels and messages.

Corresponding to the mentioned studies of price elasticity, this examination investigated the response to price increases at the population level, using Austrian aggregate consumption data. Thus, it remains unclear, how an individual Austrian smoker will react to an increase of cigarette prices (switching of brands, reducing cigarette consumption, quitting, etc.). In Mexico, an upper middle-income country, Saenz-de-Miera et al. examined the response of cigarette smokers to a tax increase at the individual level. Applying face-to-face interviews of about 45 min, the authors assessed many different smoking related perceptions and behavior before and after a 2007 cigarette tax increase (baseline and follow-up). Their results suggest, that tax increases definitely have an impact on the individual consumer: the overall self-reported cigarette price increased (12.7%), consumption decreased (29%) and the price became a more important reason to consider quitting (18).

In recent years, more and more countries introduced smoke-free laws. The WHO even states, that bans are the most widely applied policy measure (67 countries) and covered 1.8 billion people in 2020. A smoke-free environment can be effective in protecting people from the negative health effects of second-hand smoke, including cardiovascular diseases and even cancer, and in supporting smokers to stop (2, 5). Although these measures are milestones in the fight against the global tobacco epidemic, the present study indicates, that raising tobacco prices is crucial for reducing tobacco consumption. Frazer et al. analyzed 77 studies from 21 countries to assess the effects of smoking bans on health and smoking behavior. Contrary to detected positive effects on cardiovascular, respiratory and perinatal health outcomes of smokers and non-smokers, the evidence that smoke-free laws affect smoking prevalence and cigarette consumption is heterogeneous (20). Italy introduced a strict smoking ban in 2005. Shortly after the implementation data showed, that smoking prevalence decreased by 1.11% in men and 1.03% in women per year between 2004 and 2006, whereas the decline had been smaller until 2004 (0.53% in men and 0.25% in women). The smoking rate declined from 26.2% in 2004 to 25.6% in 2005 and 24.3% in 2006. Changes in smoking prevalence were only significant between 2003–2004 and 2005–2006 in men and smokers under 45 years of age. The results indicate, that the new smoking ban accelerated the decreasing smoking rate as of 2004 (21). Gualano et al. analyzed prevalence and consumption in Italy between 2001 and 2013. Smoking rates significantly declined from 28.9 to 20.6% in the observed time period, with a stronger decrease among men. A reduction in cigarette consumption was observed in almost the same manner (from 16.4 to 12.7 cigarettes per day). However, no statistically significant point of trend change associated with the introduction of the 2004 smoke-free law was found. The authors suggest to prioritize other tobacco control measures, such as price policy (22).

According to the WHO, graphic warning labels on tobacco products are the tobacco control tool with the biggest improvement since 2007. Sixty percent of the world's population were covered by this measure in 2020 (101 countries). Deterrent pictures and warning labels are important to inform current smokers about the hazards of smoking to their health and that of people surrounding them (2, 5, 23). In fact, studies show, that warning labels are able to reduce cigarette consumption as well as smoking prevalence in different countries. After the adoption of graphic warning labels in 2000 in Canada, smoking rates significantly decreased by about 12–20%, which was higher compared to previous estimates (24). However, if a society is already well-informed about the negative consequences of tobacco consumption, every attempt to further reduce smoking rates by using tools to spread information about tobacco related illness and risks could become quite difficult. Kahnert et al. assessed the implementation of the Tobacco Products Directive 2014/40/EU (TPD2) in 2016, which required packages to carry a deterrent picture, a text warning and information for quit services. Their results indicate, that the new warning labels increase salience, but do not increase self-reported cognitive or behavioral reactions (25).

In conclusion, this paper demonstrates that price policy and taxation are striking strategies to control the global tobacco epidemic and should encourage governments to use price policy and taxation more intensively.

## Data Availability Statement

The original contributions presented in the study are included in the article/supplementary material, further inquiries can be directed to the corresponding author.

## Author Contributions

All authors listed have made a substantial, direct, and intellectual contribution to the work and approved it for publication.

## Funding

Article Processing Fee will be covered by the Medical University of Vienna.

## Conflict of Interest

The authors declare that the research was conducted in the absence of any commercial or financial relationships that could be construed as a potential conflict of interest.

## Publisher's Note

All claims expressed in this article are solely those of the authors and do not necessarily represent those of their affiliated organizations, or those of the publisher, the editors and the reviewers. Any product that may be evaluated in this article, or claim that may be made by its manufacturer, is not guaranteed or endorsed by the publisher.
